# 579. Building 'Ohana: A Social Work QI Initiative from Admission to Aloha in Burn Care

**DOI:** 10.1093/jbcr/irag033.342

**Published:** 2026-04-06

**Authors:** Ashley E Honea, W Michelle Spencer, Erica Whetten, Karen J Richey, Kevin N Foster

**Affiliations:** Diane & Bruce Halle Arizona Burn Center - Valleywise Health, Phoenix, AZ; Diane & Bruce Halle Arizona Burn Center - Valleywise Health, Phoenix, AZ; Valleywise Health Medical Center, Phoenix, AZ; Diane & Bruce Halle Arizona Burn Center - Valleywise Health, Phoenix, AZ; Diane & Bruce Halle Arizona Burn Center - Valleywise Health, Phoenix, AZ

## Abstract

**Introduction:**

The transfer of six critically injured burn patients over 2900 miles required extensive coordination across multiple agencies and disciplines. Comprehensive social work support to address complex cultural needs was initiated prior to arrival and through care transition back to home. An integrated model incorporating burn survivor peer support prior to arrival and incorporating "aloha spirit" principles, was used to ensure culturally responsive care. The purpose of this QI project was to evaluate the efficacy of the model to establish evidence-based protocols for future transfers.

**Methods:**

A 7-item survey, utilizing a 5-point Likert scale, was distributed via RedCap to patients and/or family members. Four domains included social and peer support systems, cultural support and opportunities for improvement. This was followed by structured qualitative interviews that further explored their recovery journey.

**Results:**

A total of 2 patients and 1 family member participated. All respondents rated every question as "Extremely Helpful." All felt that social work was extremely helpful identifying support services. Qualitative interviews revealed that participants valued early peer support for answering questions about burn recovery processes and identified cultural integration as a key program strength. Participants reported excellent communication and coordination through the single point of contact model, while noting limited burn survivor resources and follow-up care available at home.

**Conclusions:**

While limited by sample size, this project demonstrated the effectiveness of comprehensive social work coordination with cultural responsiveness during a mass casualty transfer (MCT). The availability of immediate peer support was invaluable to loved ones and patients. Our findings provide valuable insights for developing evidence-based protocols in MCT scenarios. Establishing defined social work roles and communication processes in MCT scenarios could benefit future patients by creating more streamlined coordination and culturally responsive care.

**Applicability of Research to Practice:**

This study highlights the foundation for evidence-based protocols incorporating social work-led coordination, peer support, and cultural responsiveness in MCT scenarios, while demonstrating the value of integrating a burn survivor into the care team to facilitate smoother transitions back to communities with limited resources. The findings show how this culturally informed model with continuity of care through a single contact point improves patient experience and can be replicated in similar complex healthcare coordination challenges involving diverse patient populations.

**Funding for the study:**

N/A.

